# T2-Low Asthma: A Discussed but Still Orphan Disease

**DOI:** 10.3390/biomedicines11041226

**Published:** 2023-04-20

**Authors:** Francesca Peri, Alessandro Amaddeo, Laura Badina, Massimo Maschio, Egidio Barbi, Sergio Ghirardo

**Affiliations:** 1 Department of Medical, Surgical and Health Sciences, University of Trieste, 34127 Trieste, Italy; 2 Institute for Maternal and Child Health-IRCCS “Burlo Garofolo”, 34137 Trieste, Italy

**Keywords:** T2-low asthma, refractory asthma, non-T2 asthma, orphan disease

## Abstract

Asthma affects 10% of the worldwide population; about 5% of cases are severe with the need for target therapies such as biologics. All the biologics approved for asthma hit the T2 pathway of inflammation. T2-high asthma is classified as allergic and non-allergic, whereas T2-low asthma can be further defined as paucigranulocytic asthma, Type 1 and Type-17 inflammation and the neutrophilic form that accounts for 20–30% of all patients with asthma. Neutrophilic asthma’s prevalence is even higher in patients with severe or refractory asthma. We searched Medline and PubMed archives from the past ten years for articles with the subsequent titles: “neutrophilic asthma”, “non-type 2 asthma” and “paucigranulocytic asthma”. We identified 177 articles; 49 were considered relevant by the title and 33 by the reading of the abstract. Most of these articles are reviews (*n* = 19); only 6 are clinical trials. No study identified an effective treatment. We used the literature reported by these articles to search for further biologic treatments that target pathways different from T2. We identified 177 articles, 93 of which were considered relevant for the review and included in the present article. In conclusion, T2-low asthma remains poorly investigated in terms of biomarkers, especially as a therapeutic orphan disease.

## 1. Introduction

Asthma remains a hot topic in pulmonology medicine due to its high prevalence, impact on life quality and economic burden. Pediatric and adulthood asthma significantly differ in physiopathology and the number of patients with severe asthma needing step-up therapy to biological treatments. However, up to 7% of children have been diagnosed with asthma and fewer than 5% present with severe asthma. The latter type of asthma is a rare condition that should always be cared for at a reference center with dedicated pediatric respiratory expertise [1,2]. Asthma is even more common in adulthood, affecting more than 8% of the population with almost 10% of cases presenting with severe asthma. A lower prevalence of severe asthma seems likely in adults and children due to the difficulty distinguishing between severe and difficult-to-treat asthma. Although the difference between difficult-to-treat and severe asthma could appear poorly relevant at a first glance, it is indeed crucial for the correct management of the disease. Severe asthma is present if the patient strictly adheres to therapeutic indications beyond chronic drug administration and environmental exposure limitations.

On the contrary, the term difficult-to-treat asthma includes all patients with poor disease control primarily due to poor compliance with treatments and, as a subset, includes patients with severe asthma [3]. However, despite its low prevalence, severe asthma drains more than 60% of the total asthma costs [4,5]. Severe asthma costs have been raised due to introduction of monoclonal antibodies as part of the therapeutic options for this condition. Since the approval of omalizumab in 2003 by the Food and Drug Administration (FDA), five new biological treatments have been introduced for severe T2-driven asthma [6]. These therapies interfere with T2-inflammation in unique ways: omalizumab targets Immunoglobulin E (IgE); mepolizumab and reslizumab target interleukin 5 (IL-5); benralizumab targets IL5-receptor; and dupilumab targets IL-4 and IL-13. The last two therapies play a vital role in the switching to IgE and their production, as well as mast cell activation, Th2 differentiation, goblet cells hyperplasia and eosinophilic inflammation.

All these biologics share, as a target, T2-high inflammation. This approach neglects the well-known presence of at least four classically recognized groups of asthma inflammation: eosinophilic inflammation, eosinophilic nonallergic inflammation, paucigranulocytic inflammation and types 1 and type 17 neutrophilic inflammation [1]. Most new drugs under investigation in the pipeline are widening the spectrum by targeting interleukin (IL)-25 and IL-33 [7]. Severe asthma refractory to biologic agents seems likely to be sustained by a significant slice of T2-low inflammation characterized by different T-cell effectors (T-helper 1 and T-helper 17) and specific cytokines (IL-17, IL-6, IL-8, interferon (IFN) and tumor necrosis factor (TNF)). Sputum analysis offers another classification of T2-low asthma as neutrophilic and paucigranulocytic, depending on the neutrophil count and with no eosinophils detected in the sputum [8]. In the present review, we will discuss only T2-low asthma; we will ignore other conditions that may mimic it or should be ruled out as part of the differential diagnosis. In particular, we will not cover the excessive dynamic airway collapse, which may mimic bronco-reactivity [9], nor pneumoconiosis and professional lung diseases that may lead to bronchial neutrophilia [10].

## 2. Search Strategy

We conducted a narrative review by choosing articles based on the relevance of their contributions to the issue. Our research focused on post-2002 publications and emphasized the past 15 years. Nevertheless, we include the most referenced, relevant, and influential older publications. We searched Medline and PubMed from 2002 to December 2022 using the following terms: ‘neutrophilic asthma’, ‘non-type 2 asthma’ and ‘paucigranulocytic asthma’, and ‘severe asthma and sputum neutrophils’. We included randomized controlled trials (RCTs), observational studies, retrospective studies, reviews and case reports. To determine whether the title was pertinent to the matter, we read the abstract and, if relevance was confirmed, we evaluated the whole article. We favored recent reviews from high impact journals and randomized, double-blind, placebo-controlled studies relevant to the matter.

**Figure 1:**
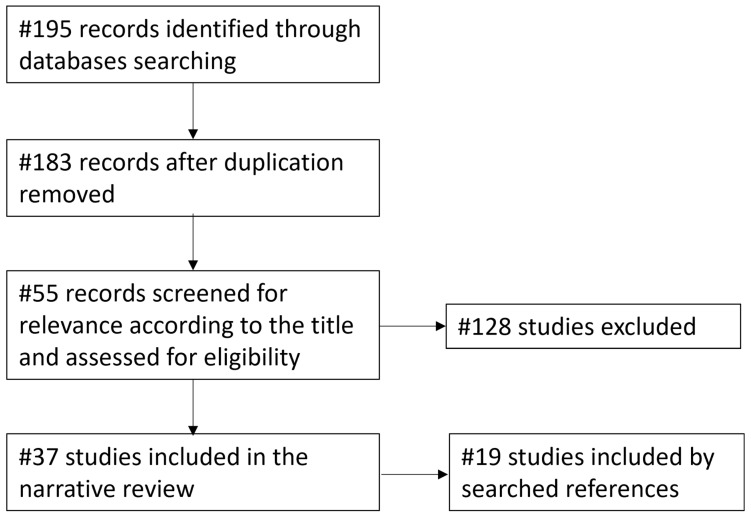
The process of selecting articles.

For ‘neutrophilic asthma’, we found a total of 65 articles, 26 of which were considered suitable by the title and 21 by the abstract; ‘non-type 2 asthma’ (total 18 articles/6 relevant titles/4 articles); ‘paucigranulocytic asthma’ (total 37 articles/11 relevant titles/6 articles), ‘severe asthma and sputum neutrophils’ (total 75 articles, 12 relevant/6 articles). In the second step, we broadened our search by selecting related articles found in the references of the above-mentioned search. The selection of the articles is resumed in Figure 1.

## 3. Results

The great majority of articles (*n* = 19) were comprehensive reviews, though none of them was a systematic review. Six clinical trials were identified by our search (Table 1). Here we discuss the most significant cornerstones of T2-low asthma.

### 3.1. Markers and Molecular Pathways

While type-2 asthma is defined by high values of exhaled nitric oxide and blood eosinophils count, T2-low asthma definition is negative. Indeed, T2-low asthma is defined by the absence of T2-high markers; therefore, T2-low has no validated biomarkers in clinical practice [20]. Nevertheless, some pathways and possible related biomarkers have been widely identified and studied in vitro and in vivo to reach potential therapeutic targets. This search would lead to better identification of the patient’s endotype with more precise therapeutic tailoring. The most frequently cited endotype method reported in the articles relies on sputum cell analysis. Sputum cytology identifies four possible patterns of airway inflammation in asthmatic patients: eosinophilic, neutrophilic, mixed granulocytic and paucigranulocytic asthma [21]. They mostly overlap with the four classically recognized groups of asthma inflammation already reported [1].

### 3.2. Innate Lymphoid Cells’ Role in T2-Low Asthma

Most of the literature exploring the role of innate immunity in asthma is focused on T2-high forms, including the innate lymphoid cell role [22]. In the T2-high forms, the core is represented by the Il-33/ST2 pathway which represents the bridge between the innate response and the adaptative T2-high immune response [23]. During the past decade, innate lymphoid cells (ILC) were categorized into five groups: ILC1 and NK, ILC2, ILC3 and lymphoid tissue inducer cells (LTi cells). ILC1-3 interactions and roles present several similarities with Th1, Th2 and Th17, respectively [24]. Therefore, for the purpose of the present review, we will focus on ILC1/NK and ILC3. In mucosal response and asthma development, ILCs seem to be key in the early phases of asthma development. Moreover, in asthma ILCs maintain memory-like knowledge of the pollutants and allergens encountered, producing a very peculiar behavior for innate immunity cells. The initial impact of pollutants on mucosal immunology seems to drive the subsequent inflammatory cascade, orienting it in a T2-low or a T2-high fashion [24]. ILC1s and ILC3s drive the epithelial inflammatory response in patients who are developing non-allergic, T2-low asthma. Upregulation of IL-1β in obese patients leads to ILC3 stimulation in the lungs, which in turn overexpress IL-17A. Similarly, pollutants lead to ILC1 and ILC3 activation. Moreover, ILC1s are crucial in obesity-related inflammation, secreting TNF-α, INF-γ and the costimulatory cytokine IL-18; the hallmark of ILC1 is the production of INF-γ because the subset of ILC1 127+ produces only INF-γ [25]. As reported, ILC3 produces IL-17A, which presumably plays a major role in chronic disease and recruiting neutrophils that release granulocyte monocytes colony-stimulating factor (GM-CSF) that sustains neutrophils and macrophages proliferation [26]. Therefore, ILC1 and ILC3 altogether are key in starting neutrophils recruiting and degranulation combined with differentiation of the macrophage in M1 type, resulting in further INF-γ releasing [27].

### 3.3. Neutrophilic Asthma

Neutrophilic asthma is mainly defined by the high prevalence of neutrophils in the sputum. Although there is still no consensus about the percentage of sputum neutrophils that identifies a patient as having a NA, a value above 60% or a neutrophil count ≥5 · 10^9^/L on at least two occasions is widely accepted [28,29]. Moreover, sputum analysis shows increased toll-like receptors (TLRs), such as TLR2 and TLR4, and several pro-inflammatory cytokines driving T2-low asthma pathobiology [8]. TLR activation determines the shift toward T helper 1 (Th1) and T helper 17 (Th17) activation with subsequent release of IFN-γ, TNF-α, as well as IL-8, IL-17A, IL-17F, and IL-22 (Th17) [14]. IL-8 is one of the main chemotactic factors induced by Th17 and acts via C-X-C motif chemokine receptor 2 (CXCR1) and CXCR2, together with leukotriene B4 (LTB4) [30]. GROα, CXCL10 and CCL2 are also overexpressed in the sputum [31]. In turn, recruited neutrophils secrete IL-8, creating a positive feedback loop and promoting further recruitment and neutrophilic inflammation [8]. Dysregulation of TNF is reported with an increase in its receptors tumor necrosis factor receptor 1 and 2 (TNFR1 and TNFR2) in the sputum of patients with neutrophilic asthma subtypes compared to non-neutrophilic asthmatic patients [13]. Therefore, as suggested by this cytokinin expression pattern, the role of innate immunity in neutrophilic asthma appears to be much more relevant than the adaptive role for allergic eosinophilic asthma [32].

Some studies discussed whether T2-low asthma, especially neutrophilic asthma, is a proper subtype or the consequence of severe eosinophilic asthma treated with steroids [33]. While promoting eosinophils apoptosis, steroids inhibit neutrophils apoptosis, leading to an increase in airway neutrophilia [34,35]. Nevertheless, even if steroids seem to prolong neutrophils’ survival in the airway epithelium, this effect cannot be blamed for neutrophils homing into the bronchi. Moreover, from a clinical perspective, neutrophilic inflammation in asthmatic patients has been observed regardless of steroid therapy as well as in steroid-naïve patients [36,37]; cytokines frequently involved in T2-low asthma, such as IL-17A and IL-22, are often resistant to steroid action [8].

Neutrophils in the sputum are not just a marker of the endotype but also a prognostic marker because, in neutrophilic asthma (NA), a high percentage of sputum neutrophils is inversely proportional to forced expiratory volume in one second-forced vital capacity ratio (FEV1/FVC) [19,21] and pre- and post-bronchodilator predicted values of FEV1 [38].

### 3.4. Paucigranulocytic Asthma

Paucigranulocytic asthma is less frequently found and characterized by the absence of both eosinophilic and neutrophilic inflammation, making it a distinct non-inflammatory asthma subtype [39]. Indeed, there is evidence of airway hyper-responsiveness secondary to structural abnormalities involving airway smooth muscle, abnormal response to neuronal activation and neuronal activation [8]. This subtype of asthma is characterized by high airway responsiveness to methacholine, smooth muscle proliferation and hyperplasia and subepithelial matrix deposition, leading to fibrosis and mucus in the nearly complete absence of airway inflammation [39,40]. Several cytokines and pollutants trigger muscle contractility or alter calcium signaling [40]. Animal studies suggest nerve growth factors may be involved in bronchial hyper-reactivity and airway remodeling [41,42]. Lastly, some authors suggest that paucigranulocytic asthma should still be considered as a low-grade inflammatory asthma subtype with a low number of eosinophils, blood leukocytes and blood eosinophils [43].

We will not cover eosinophilic inflammation and eosinophilic nonallergic inflammation because both share a T2-high inflammation.

### 3.5. Mixed Granulocytic Asthma

Mixed granulocytic asthma is characterized by both eosinophilic and neutrophilic asthma, with the concurrency of more than 40% of neutrophils and more than 3% of eosinophils in the sputum [32]. Therefore, these patients can be classified either as having neutrophilic or eosinophilic asthma. However, they should likely be classified apart due to the complex interaction between these two forms of inflammation (T2-driven and T2-low driven inflammation). These patients present blood eosinophilia and a lower prevalence of gastroesophageal reflux disease than patients with neutrophilic asthma. In mixed granulocytic asthma, the neutrophilic component appears more relevant in determining the prognosis due to the poor response to ICS. Corticosteroids are usually effective against the eosinophils and can lead to eosinophil percentage decrease in such patients, leading to their reclassification as having neutrophilic asthma [44]. Mixed granulocytic asthma is also often found in smokers [45].

### 3.6. Environmental Triggers

**Figure 2:**
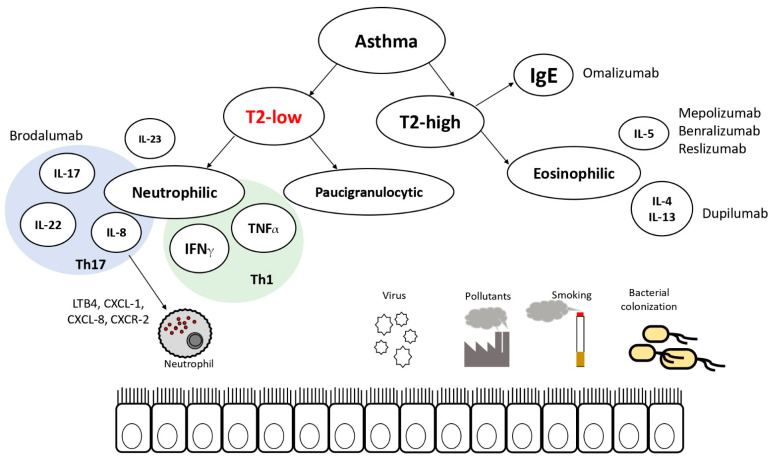
Pathogenesis of T2 high and T2-low asthma: IL: interleukin; TNF: tumour necrosis factor; INF: interferon; IgE immunoglobulin E; LTB4: leukotriene B4, CXCL: chemokine (C-X-C motif) ligand, CXCR: C-X-C motif receptor.

Unlike allergic asthma, T2-low asthma reflects an immunological innate and non-specific response to environmental factors and infectious agents rather than an adaptive response to allergens [19,33]. T2-low asthma is more prevalent in countries with higher levels of airway pollutants characterized by pathogen-associated molecular patterns (PAMPs) that activate macrophages in the airway epithelium and lead to the release of TLRs, IL-8 and IL-6 and subsequent pulmonary neutrophil recruitment [33,46,47]. The PAMPs TLRs cascade also triggers NLR Family Pyrin Domain Containing 3 (NLRP3) activation with IL-1β release and consequent inflammation and pyroptosis l [48]. Smoking is another strong environmental trigger of macrophage activation and production of IL-17A, IL-6 and IL-8, leading to airway neutrophilia in asthma patients [47]. Smokers usually have severe asthma with poor control of symptoms and poor response to inhaled corticosteroids [49]. This issue could be partially explained as a consequence of the neutrophilic inflammation of the airway being more expressed by these patients, making this form of asthma more challenging to control than the allergic one (Figure 2).

### 3.7. Clinical Phenotype

Middle-aged obese females represent the most frequent phenotype with underlying T2-low endotype asthma; their risk of having this endotype is even increased if they present a history of prolonged inhaled corticosteroids (ICS) treatment [18,33,50,51,52]. Several studies showed that obese patients with asthma presented a sputum inflammatory pattern characterized by increased IL-17A expression and a higher percentage of neutrophils in sputum than non-obese asthmatic patients [53]. Upregulation of the nucleotide-binding domain and leucine-rich repeat-containing proteins (NLRP3) inflammasome has been identified as a trigger of neutrophilic inflammatory response in patients with obesity-related asthma [54]; it has been associated with a systemic inflammatory response [55]. Patients with obesity-related non-atopic asthma present an increased INF signature at the RNA sequence of the CD4+ Lymphocytes. This activation of the interferon pathway in obese patients is, overall, overlapped with the viral response one [56]. Obese patients present increased cytolysis with higher circulating free fatty acids and other damage-associated molecular patterns (DAMPs) that lead to non-specific inflammation through the NLRP3/IL-1β cascade. Therefore, NLRP3 overactivation by DAMPs can be addressed for part of the higher neutrophilic asthma risk experienced by obese patients, together with deconditioning that led to higher bronchial responsiveness. It is widely known that insulin resistance and systemic inflammation worsen the association between asthma and obesity [57]. These patients usually present with a history of repeated exacerbations. A growth of IL-1ß following previous exacerbations has been associated with increased IL-6 and systemic inflammation, thus triggering a vicious cycle. Finally, IL-6 and the above-mentioned IL-8, by recruiting neutrophils to the airways, enhance inflammation and cause airway remodeling with thickened bronchi, impairing bronchodilator responsiveness. Besides obesity related T2-low asthma, most authors recognize two other phenotypes of patients with T2-low asthma: smokers and non-atopic, very late onset paucigranulocytic or neutrophilic asthma. High neutrophil count in the sputum is often characterized in smoking-related asthma. That condition is likely caused by PAMPs and oxidative stress with the consequent persistence of innate inflammatory response [45]. Non-atopic, very late onset asthma is defined as a diagnosis of asthma established for the first time after age 50 or 65 years in the absence of atopy [58]. This particular form of asthma is described as the consequence of the mechanical senescence of bronchial and parenchymal [59]. Moreover, ageing leads to reduced peripheral tolerance for Th1, Th-17 and NLRP3/IL-1ß with paucigranulocytic or neutrophilic asthma [60,61].

### 3.8. Pathogenes

Eosinophilic asthma is not associated with bacteria colonization outside superimposed acute respiratory infections [62]. Conversely, an increased prevalence of Hemophilus influenzae and Moraxella catarrhalis is found by culturing the sputum of patients with neutrophilic asthma, configuring chronic bacterial colonization. The prevalence of bacterial isolation and the number of colonies forming unit (CFU) is strictly related to neutrophils levels, as seen in other chronic respiratory conditions, such as bronchiectasis and chronic obstructive pulmonary diseases COPD [63,64]. Moreover, neutrophilic asthma shares with bronchiectasis the lack of response to inhaled cortico-steroids (ICS) and the beneficial effect of azithromycin, as shown below. It may be speculated that high doses of inhaled steroids contribute to airway infection due to local immune system suppression and subsequent airway neutrophilia. *H. influenzae*, *M. catarrhalis*, and *S. Aureus* can induce a Th17 response, maintaining chronic airway neutrophilia [65]. Lastly, as mentioned above, the prevalence of T2-low asthma increases with age and is probably sustained by the vicious cycle between bacterial infections, neutrophil recruitment and inflammation that leads to airway remodeling and bronchiectasis. The same bacteria *H. influenzae* and M catarrhalis adding S. pneumoniae are reported as the first three bacteria involved in developing protracted bacterial bronchitis (PBB) in childhood. PBB represents the first reversible cause of chronic cough in toddlers; its persistence is a significant risk factor for developing bronchiectasis. On the other hand, PBB does not correlate with asthma risk [66].

### 3.9. Pediatric T2-Low Asthma

The role of neutrophils in childhood asthma is still partly unclear. Firstly, the main barrier to pursuing the research is related to difficulties in adequate sampling of the lower airway of this population. One of the few prior observational studies showed a prevalence of neutrophilic asthma in almost 30% of stable asthmatic adults and 20% of stable asthmatic children [67]. A future proposal is implementing in vitro methodologies, using peripheral blood from children to study neutrophil function and migration in various lung environments, as shown by Grunwell et al. [68], that suggest impairments in neutrophils function in such patients. However, the great effort of tracking airway neutrophilia might be overtaken by the need for more clarity around its role in promoting T2-low bronchial inflammation in children with severe asthma. For example, in one study children with severe refractory asthma presented with an increased sputum neutrophils count [69]; these data were not confirmed in three other studies conducted on bronchoalveolar lavage [70,71,72]. Lastly, an increased count of intraepithelial neutrophils in bronchial biopsies of children with severe asthma was associated with better lung function and fewer exacerbations [73]. Moreover, studies in children were gathered from the severe asthma cohorts, excluding those with mild-to-moderate disease. Despite elevations in severely asthmatic children with a pronounced pro-inflammatory endotype, no difference in clinical characteristics was observed between children with high or low neutrophil counts [74].

Analysis of bronchoalveolar lavage (BAL) in children with refractory neutrophilic asthma revealed a cytokine pattern consistent with a mixed Th17/Th1/Th2 response, similar to adults with neutrophilic asthma. In particular, a strong association was detected with cytokines responsible for neutrophil chemotaxes, such as CXCL8, CXCL10 and TNF-α, as well as Th17 differentiation (IL-6) and expression (IL-17) [75]. The following points should be noted: (1) children were on ICS high dose and many also took oral corticosteroids, leading to a reduction in peripheral blood eosinophils count; and (2) viruses and bacteria were detected mainly in patients with BAL neutrophils presence but no clinical evidence of infection. Another recent study found a positive correlation between neutrophil levels in the sputum and IL-8 and IL-17 in sputum in childhood neutrophilic asthma, strengthening the link between Th17-immunity and the pathogenesis of neutrophilic asthma [76].

### 3.10. Therapies and Targets

#### Randomized Controlled Trials

The great effort in finding promising therapeutic approaches for asthma has been far more unsatisfactory in T2-low asthma than in eosinophilic asthma. Target treatments demonstrating efficacy in the T2 pathway failed to achieve results in the T2-low test. The only approved drug class for neutrophilic asthma is macrolides; approval was secured via a randomized controlled study called AMAZES and published in 2020, which compared azithromycin 500 mg taken three times per week to placebo in 420 adults with asthma resistant to medium-to-high dose corticosteroids plus long-acting β2-agonist. This study ascertained azithromycin’s effectiveness in lowering the number of exacerbations, improved quality of life overall, and reduced TNF and its receptors in the sputum of patients with neutrophilic asthma. The mechanism of action is still unknown, but it could probably be headed to macrolides’ well-known anti-bacterial and anti-inflammatory effects in other conditions, such as cystic fibrosis and bronchiectasis. As a complementary study to the AMAZES authors published the effect of azithromycin on the airway microbiota. It was found that in the sputum of the 61 patients tested, azithromycin led to a reduction only in the Hemophilus load with no effect on the overall bacterial load; moreover, azithromycin led to antimicrobial resistance against macrolides and, in a minority of cases, tetracyclines [77,78,79,80]. The most examined target in the pre-clinical investigation was CXCR2, which is also known as IL8 receptor beta (IL8-RB); CXCR2 has a crucial role in promoting neutrophil migration to the airways. Two studies targeted CXCR2 in human subjects, focusing on neutrophilic inflammation. Nair et al. randomized 34 patients with neutrophilic asthma to receive a CXCR2 antagonist (SCH527123) or a placebo. SCH527123 administration reduced one-third of neutrophils in the sputum of patients with severe asthma, which was the study’s primary endpoint. This analysis noted a slight reduction in mild exacerbations (1.3 vs. 2.25 over 4 weeks) [15]. Another CXCR2 antagonist (AZD8309) was tested on twenty healthy volunteers before administration of LPS by inhalation (lipopolysaccharide), a potent inducer of neutrophilic airway response. Pre-emptive AZD8309 administration resulted in a 77% reduction in sputum cellularity and a similar reduction in neutrophils [16]. In 2016, these studies led to a clinical investigation using AZD5069, an antagonist of CXCR2, to find a dose RCT. The authors randomized four groups of adults with uncontrolled asthma despite medium-to-high dose ICS plus long acting β2 agonists. Three groups received three different doses of AZD5069, while one received a placebo. Over six-months, all three groups of patients treated with AZD5069 regardless of the dose indicated a higher number of exacerbations than the placebo group, although not to a statistically significant level. However, the authors defined neutrophilic asthma despite the absence of appropriate biological markers to endotype patients before enrollment, mainly because second-line treatment-resistant asthma necessarily implies a significative neutrophilic component [17]. The leukotriene pathway was also targeted, using a 5-lipoxygenase-activation protein inhibitor (GSK2190915). GSK2190915 revealed no efficacy in reducing smear neutrophils count despite the achieved suppression of leukotriene4 (LTB4) [19]. In asthmatic patients out of background treatment, GSK2190915 demonstrated effectiveness of a similar level to montelukast and a trend of being inferior to medium-dose ICS [81].

Several other biomarkers have been targeted in the past few years for treating asthma with little or no clinical efficacy. We will now walk through the few trials conducted on this subject. Golimumab, an anti-TNF-α monoclonal antibody, was assessed in treating persistent asthma despite high doses of ICS. In this trial, adult patients were randomized to receive a placebo or three doses of Golimumab regardless of patients’ endotypes. Treatment resulted in a higher incidence of malignancy in the Golimumab group, the absence of benefit in terms of FEV1 and severe exacerbation, leading to study interruption after 24 weeks [11]. The lack of efficacy of another anti-TNF-α drug, the soluble receptor Etanercept, was proven in a randomized controlled trial conducted on adults with moderate-to-severe asthma already taking high doses of ICS; the trial proved no difference compared to the placebo over 12 weeks of administration [12]. Another randomized controlled trial was designed to assess the usefulness of anakinra, an IL1 receptor blocker, as rescue therapy in adults with mild allergic asthma or allergic rhinitis. This trial excluded patients without allergic sensitization to dust mites or cat epithelium. The trial started in 2019 but was stopped before its conclusion due to the COVID-19 pandemic. The authors focused their attention on the role of IL1 in eosinophilic recruiting. However, the IL1 cytokine superfamily plays a key role beyond this pathway in the innate immune system; it contributes to neutrophils infiltration and pyroptosis and is crucial in the inflammasome. Interestingly, IL1 was correlated with both types of inflammation (neutrophilic and eosinophilic) in both asthma and COPD [82].

### 3.11. Promising Drugs

Lastly, we cite a recent bench model and a case report that seems to be promising. The bench model treats Fezakinumab, an anti-IL22 monoclonal antibody that downregulates the inflammatory genes signature on blood, sputum and even bronchial brushing of patients with mild-to-severe asthma [83,84]. The case report concerns a 56-year-old asthmatic female treated with ustekinumab (anti-IL-23 and IL-12 monoclonal antibody) for psoriasis. She presented a complete remission of psoriasis and asthma, leading to a marked enhancement of exercise tolerance. We agree with the author’s suggestion that, presumably, ustekinumab quenched this patient’s Th17-driven component of bronchial inflammation. She was poorly responsive to ICS and needed four-to-six courses of oral corticosteroids (OCS) per year for her asthma before ustekinumab and none afterwards, with other asthma background treatments and any need for albuterol treatment permanently interrupted [85].

### 3.12. Physical Therapy and Thermoplasty

Regardless of the underlying cause, asthma-related bronchial responsiveness improves upon performing repeated short submaximal exercises regularly and, overall, with training. The literature usually refers to this complex interaction between physical effort and asthma using the term “sports therapy” which, in the appropriate context can significantly improve exercise tolerance and quality of life [86]. Thermoplasty is a bronchoscopic procedure in which a probe, sequentially introduced in each segmental bronchus, delivers a circumferential thermal shock to the bronchial wall. This procedure leads to the disruption of smooth cells, the fibrous substitution of the bronchial wall and the abolition of bronchial responsiveness regardless of the underling stimulus. This procedure is invasive, time-consuming and expensive, requiring at least two separate bronchoscopy sections for each patient; it is also riskier than a standard bronchoscopy. Therefore, proper patient selection is mandatory before proceeding with thermoplasty. Patients with severe neutrophilic inflammation should be considered for this procedure due to the lack of other equally effective therapeutic options [87].

### 3.13. Limits of the Available Evidence

None of the trials that focused on clinical efficacy were explicitly designed for T2-low asthma or differentiated the subjects enrolled on its endotype, which would have allowed a posteriori analysis. Due to the lack of endotyping before administering the investigated drug, most of these trials may be considered missed opportunities in searching for a promisingly effective drug for T2-low asthma. There is a need to change the mindset of trial design for new treatments for patients with ICS-resistant asthma. All these trials imply a simple baseline for endotyping patients, allowing more precise data analysis and leading to much more personalized drug choice. Indeed, ineffective drugs could have statistically significant effects if administered to patients with a specific endotype. These aspects should be considered relevant because 5–10% of asthmatic patients are steroid-resistant and the chances of such subjects having neutrophilic inflammation are much higher [82]. Moreover, risk of hospitalization in patients with more than 60% of neutrophils in the sputum doubles [33]. For these reasons, we suggest the need for endotype-guided trials, only enrolling patients with a high neutrophil count in their sputum. Lastly, we should remember that patients with neutrophilic asthma and bronchiectasis share the same airway microbiology and part of immune system activation [14,88]. Even the opportunity to target neutrophils is a doubtful benefit without endotype-driven trials on humans [89]. The efficacy of an approach focused on targeting a single chemoattractant of the neutrophils could also be ineffective due to the vast complexity of the neutrophils recruiting cascade [31], that is, currently expanding even further, including neutrophil extracellular traps (NETs) [90,91]. Moreover, the effect of steroids on airway neutrophils should be investigated further to understand if there is a pathogenetic role for such drugs in neutrophil activation/migration in the bronchi [36]. The impact of ICS on neutrophilic asthma remains one of the main unresolved issues. There are suggestions that neutrophilic asthma may represent a corticosteroid resistant form of the disease rather than being a consequence of long-term steroid treatment. However, these considerations are supported by early-2000s studies on limited samples of patients [37]. We believe that low-T2 asthma represents a form of steroid-insensitive asthma engaged by innate immunity response; it has a substantially different T lymphocyte activation subset with other molecular pathways compared to T-2 high asthma.

## 4. Conclusions

T2-low asthma is often a severe disease and is poorly or non-responsive to corticosteroids even at high doses, consequently severely impacting patients’ activities and overall prognosis. Correctly identifying the endotype of the patient could be highly informative for second-line treatment-refractory patients. We suggest endotyping patients before starting biological drugs to avoid wasting time and costs. Neutrophilic asthma accounts for most T2-low asthma patients, while sputum analysis can represent a relatively easily available biomarker to detect sputum neutrophilia. Therefore, in the absence of a shared T2-low asthma definition, we suggest, in the first instance, the need for therapeutic RCT in patients with neutrophilic asthma, possibly including pediatric patients.

## Figures and Tables

**Table 1:** Targeted treatment for neutrophilic asthma.

Main Study (and Related Articles)	Year	Biomarkers	Treatment	Conclusion
Wenzel [11]	2009	TNF	Golimumab	No clinical efficacy
Holgate (Howarth, Berry, Morjaria) [12]	2011	TNF	Etanercept	No clinical efficacy
Niessen (Gibson, Brusselle, Taylor) [13]	2020	TNF	Macrolides (azithromycin)	Dysregulated TNF in pt. with NA is suppressed by azithromycin
Simpson [14]	2008	Unknown	Macrolides (clarithromycin)	Modulation of airways neutrophil accumulation and activation
Brusselle [7]	2013	Unknown	Macrolides (azithromycin)	Reduction in severe exacerbation
Nair [15]	2012	CXCR2	CXCR2 receptor antagonist (SCH527123)	Reduction in sputum neutrophils in patients with SA and sputum neutrophils
Leaker [16]	2013	CXCR2	CXCR2 antagonist (AZD8309)	Inhibition of LPS-induced inflammation
O’Byrne (Watz) [17]	2016	CXCR2	CXCR2 antagonist (AZD5069)	Reduction in mucosal, sputum and blood neutrophils without clinical efficacy
Busse [18]	2013	IL-17	Anti-IL-17R (Brodalumab)	No clinical efficacy
Chaudhuri (Follows) [19]	2014	5-lipoxygenase-activating protein (FLAP)	FLAP inhibitor (GSK2190915)	No effect on sputum cell counts or clinical endpoints in patients with asthma and sputum neutrophils

TNF tumor necrosis factor; CXCR2 C-X-C motif chemokine receptor 2; IL-17 interleukin 17; anti-IL-17R anti-interleukin 17 receptor; FLAP lipoxygenase-activating protein.

## Data Availability

Not applicable.
